# The Impact of Social Media on the Physical and Mental Well-Being of Medical Students During the COVID-19 Pandemic

**DOI:** 10.1007/s44197-023-00164-7

**Published:** 2023-11-13

**Authors:** Raaina Mahevish, Aisha Khan, Hareem Rashid Mahmood, Sadia Qazi, Hana M. A. Fakhoury, Hani Tamim

**Affiliations:** 1 College of Medicine, Alfaisal University, Riyadh, Saudi Arabia; 2 Department of Internal Medicine, American University of Beirut Medical Center, Beirut, Lebanon

**Keywords:** COVID-19, Social media, Mental health, Diet, Sleep patterns, Physical activity

## Abstract

COVID-19 pandemic has increased social media engagement globally. This study examined the correlation between social media use and physical/mental health among university students, considering gender and academic year. Out of 146 responses, 119 were analyzed after excluding participants with pre-existing psychological conditions. Results showed a significant correlation between social media use and mental health for all participants (correlation coefficient = 0.30, *p* < 0.001), indicating a negative impact on mental health with increased use. Gender-specific analysis revealed a non-significant correlation among males (*p* = 0.21), while females exhibited a significant correlation (correlation coefficient = 0.32, *p* = 0.01), suggesting an adverse effect on their mental health. Regarding physical health, females displayed an even higher correlation (correlation coefficient = 0.40, *p* < 0.001), highlighting the negative influence of social media on their physical well-being. Conversely, no significant correlation was observed among males. Analyzing by academic year, both pre-clerkship and clerkship students showed a significant correlation between social media use and mental health (correlation coefficients of 0.26, *p* = 0.01, and 0.42, *p* = 0.03, respectively). Similarly, a significant correlation was found between social media use and physical health among pre-clerkship students (correlation coefficient = 0.34, *p* = 0.001), but not among clerkship students. In conclusion, this study provides evidence of the adverse impact of social media use on physical and mental health among university students, particularly among females and across different academic years. These findings underscore the importance of promoting healthy social media habits and raising awareness about the potential negative effects on well-being.

## Background

The emergence of coronavirus (COVID-19) in December 2019 in China marked the beginning of a global health crisis [1]. World Health Organization (WHO) swiftly identified the causative agent as novel coronavirus (2019-nCoV) and declared it a public health emergency of international concern [2]. To combat the transmission, stringent prevention and lockdown measures were implemented worldwide, with Saudi Arabia being among the leading countries in addressing the crisis. During the initial months of 2020, extensive lockdown measures were enforced globally, resulting in the closure of educational institutions and a shift towards e-learning [3]. Consequently, the reliance on technology, including smartphones, escalated as people sought to stay connected. Research indicates an increase in social media use during periods of social distancing [4]. Social media platforms serve as vital sources of information and social support, particularly for individuals experiencing stress or anxiety, as face-to-face interactions are restricted during crises [4]. However, past studies have demonstrated the adverse effects of compulsive social media use on physical and mental health, including cardio-metabolic health, sleep patterns, self-esteem, overall well-being, and functioning, particularly among adolescents [5]. WHO has also cautioned against social media rumors, which can provoke panic, stigma, and irrational behavior. Social media play a huge impact on a student’s mental and physical health [6]. Numerous studies have focused on the impact of social media during the time of the pandemic [4, 7–12]. A study in Iraqi Kurdistan highlighted social media's significant role in spreading fear and panic, potentially adversely affecting mental health and well-being [7]. Additionally, stringent lockdown measures in Australia were linked to a notable negative impact on emotional and physical well-being [13]. In India, during the COVID-19 lockdown, increased social media use led to disruptions in mood, disturbed sleep patterns, reduced physical activity, and adverse effects on academic performance [10]. Similarly, research has delved into gender differences concerning both physical and mental health. For instance, a study in Switzerland explored these demographics [8].

Unfortunately, there is scarcity of research examining perception and impact of social media during the pandemic, specifically within the Kingdom of Saudi Arabia. Therefore, this study aims to investigate the effects of social media use on physical and mental health of medical students in a private institution in Saudi Arabia during the COVID-19 pandemic.

## Methods

### Study Design and Questionnaire Development

This cross-sectional study was conducted between February and March 2021. A customized questionnaire was developed by drawing on validated studies [14, 15] and tailored to the specific objectives and scope of our research. To ensure clarity and comprehensibility, a pilot study was carried out, involving randomly selected participants who provided feedback on the survey. Necessary adjustments were made based on their input.

The research team developed a self-administered questionnaire consisting of a consent form, demographic information, and 39 main questions across six categories: general information about COVID-19, social media use, mental health, diet, sleep patterns, and physical activity. The majority of questions were presented in a Likert scale format, ranging from "Strongly Disagree" to "Strongly Agree." Data analysis was conducted by categorizing responses based on gender (males and females) and academic year (pre-clerkship: years 1–3 and clerkship: years 4–5). The general information section included seven main questions (Table 1), with correct answers coded as "Agree" or "Strongly Agree." Social media use comprised seven main questions (Table 2), with correct answers similarly coded. Their use was determined based on preoccupancy, duration, and attempts to curb down. They were further assessed based on a variety of reasons for using social networking sites, such as sharing new ideas, creating a social identity and relief from academic stress among many others. Mental health assessment involved questions related to participants' interests, changes in sleep and appetite patterns, concentration issues, feelings of depression, tiredness, failure, and restlessness (Table 3). Diet assessment included questions rating eating habits, regular intake of carbonated drinks, fast food, snacks, sweets, beverages, dairy products, and meat or fish (Table 4). Seven questions were presented on different scales, with scores calculated for each participant. The scores were added for each participant and a total score ranging from 0 to 17 indicated ideal or fair eating habits, while 18–35 represented good or excellent habits. Sleep patterns were evaluated through questions regarding the time taken to fall asleep, frequency of waking up during the night, and time taken to fall asleep again (Table 5). Among the five main questions, two had subdivisions, resulting in a total of 18 questions using different scales. Physical activity was assessed by asking participants about various activities, such as running and walking, more than a mile (Table 6). Two main questions were included, with one question having subdivisions, leading to a total of eight questions. Overall physical health was determined by combining responses from the diet, sleep patterns, and physical activity sections, resulting in a total of 27 questions.

**Table 1:** Comparison of general knowledge about COVID-19 among males and females in pre-clerkship and clerkship years

Variables	Males *n* (%)	Females *n* (%)	Pre-clerkship *n* (%)	Clerkship *n* (%)
Incubation period of COVID-19 is 5–14 days	44 (97.8%)	72 (97.3%)	89 (97.8%)	27 (96.4%)
COVID-19 is transmitted				
By infected persons	45 (100%)	71 (95.9%)	89 (97.8%)	27 (96.4%)
By droplets in air	40 (88.9%)	65(87.8%)	78 (85.7%)	27 (96.4%)
By droplets on surfaces	36 (80.0%)	59 (79.7%)	74 (81.3%)	21 (75%)
By cough and sneeze	44 (97.8%)	72 (97.3%)	90 (98.9%)	26 (92.9%)
By exhalation	31 (68.9%)	42 (56.8%)	57 (62.2%)	16 (57.1%)
COVID-19 has				
Dry cough	39 (86.7%)	65 (87.8%)	79 (86.8%)	25 (89.3%)
Sore throat	37 (82.2%)	62 (83.8%)	76 (83.5%)	23 (82.1%)
Stomach pain	13 (28.9%)	20 (27.0%)	23 (25.3%)	10 (35.7%)
Diarrhea	15 (33.3%)	35 (47.3%)	36 (39.6%)	14 (50%)
Nausea	25 (55.6%)	37 (50.0%)	49 (53.8%)	13 (46.4%)
Fever	43 (95.6%)	71 (95.9%)	87 (95.6%)	27 (96.4%)
Muscle pain	36 (80.0%)	59 (79.7%)	71 (78%)	24 (85.7%)
Fatigue	42 (93.3%)	71 (95.9%)	85 (93.4%)	28 (100%)
COVID-19 can be prevented by				
Wearing mask	43 (95.6%)	71 (95.9%)	86 (94.5%)	28 (100%)
Washing hands for 20 s	39 (86.7%)	66 (89.2%)	80 (87.9%)	25 (89.3%)
Having good immune system	35 (77.8%)	59 (79.7%)	74 (81.3%)	20 (71.4%)
Balanced nutrition	29 (64.4%)	51 (68.9%)	62 (68.1%)	18 (64.3%)
Vaccine	41 (91.1%)	60 (81.1%)	79 (86.8%)	22 (78.6%)
COVID-19 mortality rate is higher in elderly	45 (100.0%)	71 (95.9%)	88 (96.7%)	28 (100%)
No drug treatment available for COVID-19	30 (66.7%)	45 (60.8%)	55 (60.4%)	20 (71.4%)
COVID-19 patient needs ventilator to survive	29 (64.4%)	49 (66.2%)	59 (64.8%)	19 (67.9%)
Mean score ± STD	78.9 ± 11.8	78.2 ± 11.2	78.2 ± 11.1	79.2 ± 12.3
*p* value	0.75		0.69	

Data reflect the participants who agreed to the corresponding question

**Table 2:** Comparison of social media use during the COVID-19 lockdown among males and females in pre-clerkship and clerkship years

Variables	Males *n* (%)	Females *n* (%)	Pre-clerkship *n* (%)	Clerkship *n* (%)
Do you feel preoccupied with the internet?	33 (73.3%)	62 (83.8%)	73 (80.2%)	22 (78.6%)
Do you feel the need to use the internet with increasing amounts of time in order to achieve satisfaction?	28 (62.2%)	47 (63.5%)	59 (64.9%)	16 (57.1%)
Have you repeatedly made unsuccessful efforts to control, cut back, or stop internet use?	27 (60.0%)	49 (66.2%)	56 (61.5%)	20 (71.4%)
Do you feel restless, moody, depressed, or irritable when attempting to cut down or stop internet use?	27 (60.0%)	49 (66.2%)	60 (65.9%)	14 (50%)
Do you stay longer than originally intended?	38 (84.4%)	64 (86.5%)	79 (86.8%)	23 (82.1%)
Do you use the internet as a way of escaping from problems or of relieving a dysphoric mode (e.g., feelings of helplessness, guilt, anxiety, depression)?	22 (48.9%)	39 (52.7%)	48 (52.7%)	13 (46.4%)
I use social networking sites to				
Keep in touch with my relatives	26 (57.8%)	49 (66.2%)	60 (65.9%)	15 (53.6%)
Share new ideas	14 (31.1%)	32 (43.2%)	34 (37.4%)	12 (42.9%)
Create my social identity	18 (40.0%)	24 (32.4%)	32 (35.2%)	10 (35.7%)
Get information regarding current social events and news	40 (88.9%)	66 (89.2%)	81 (89%)	25 (89.3%)
Do research work	15 (33.3%)	52 (70.3%)	51 (56%)	16 (57.1%)
For group/collaborative learning	18 (40.0%)	54 (73.0%)	57 (62.6%)	15 (53.6%)
To seek help from my professors	13 (28.9%)	38 (51.4%)	41 (45.1%)	10 (35.7%)
To learn about my curricular aspect	24 (53.3%)	52 (70.3%)	58 (63.7%)	18 (64.3%)
To get relief from academic stress	38 (84.4%)	65 (87.8%)	78 (85.7%)	25 (89.3%)
Mean score ± STD	56.3 ± 22.4	66.8 ± 20.0	63.5 ± 20.8	60.5 ± 23.7
*p* value	0.01		0.51	

The data reflects the participants who agreed to the corresponding question

**Table 3:** Comparison of mental fitness during the COVID-19 pandemic among males and females in pre-clerkship and clerkship years

Variables	Males *n* (%)	Females *n* (%)	Pre-clerkship *n* (%)	Clerkship *n* (%)
Little interest or pleasure in doing things	18 (40.0%)	28 (37.8%)	37 (40.7%)	9 (32.1%)
Feeling down, depressed, or hopeless	13 (28.9%)	23 (31.1%)	31 (34.1%)	5 (17.9%)
Changes in sleep patterns	22 (48.9%)	49 (66.2%)	55 (60.4%)	16 (57.1%)
Changes in appetite patterns	17 (37.8%)	40 (54.1%)	45 (49.5%)	12 (42.9%)
Feeling tired or having little energy	20 (44.4%)	46 (62.2%)	51 (56%)	15 (53.6%)
Feeling bad about yourself or that you are a failure or have let yourself or your family down	16 (35.6%)	38 (51.4%)	41 (45.1%)	13 (46.4%)
Trouble concentrating on things, such as watching television or studying	18 (40.0%)	43 (58.1%)	45 (49.5%)	16 (57.1%)
Have you been moving or speaking so slowly that it is noticeable?	3 (6.7%)	10 (13.5%)	11 (12.1%)	O2 (7.1%)
Being so fidgety or restless that you have been moving around a lot more than usual	8 (17.8%)	24 (32.4%)	24 (26.4%)	8 (28.6%)
Mean score ± STD	33.3 ± 29.1	45.2 ± 29.0	41.5 ± 30.0	38.1 ± 28.1
*p* value	0.03		0.59	

Data reflect the participants who agreed to the corresponding question

**Table 4:** Comparison of diet during the COVID-19 pandemic among males and females in pre-clerkship and clerkship years

Variables	Males *n* (%)	Females *n* (%)	Pre-clerkship *n* (%)	Clerkship *n* (%)
How would you rate your overall habits of eating healthy foods?	43 (95.6%)	73 (98.6%)	88 (96.7%)	28 (100%)
How many regular soda, sweet tea, juice, energy/sports drinks, sweetened-coffee or other sugar sweetened beverages did you drink each day?				
How many times a day did you eat fast/fried food/or packaged snacks high in fat/salt/or sugar?				
How many times a day did you eat regular (not low-fat) snack chips or crackers?				
How many times a day did you eat sweet foods (not the low-fat kind) or desserts, like chocolate or ice cream, and other sweets?				
How many times a day did you eat dairy products (milk, unsweetened yogurt, low-fat cheese)?				
How many times a day did you eat meat/fish/beans?				
Mean score ± STD	95.6 ± 20.8	98.7 ± 11.6	96.7 ± 18.0	100.0 ± 0.0
*p* value	0.37		0.08	

Data reflect the participants who scored a total between 18 and 35

**Table 5:** Comparison of sleep during the COVID-19 pandemic among males and females in pre-clerkship and clerkship years

Variables	Males *n* (%)	Females *n* (%)	Pre-clerkship *n* (%)	Clerkship *n* (%)
On average how long does it take you to fall asleep? (^a^> 10 min)	30 (66.7%)	56 (75.7%)	66 (72.5%)	20 (71.4%)
Do you do anything in bed to help you to get to sleep, such as				
Relaxation exercises	9 (20.0%)	21 (28.4%)	25 (27.5%)	5 (17.9%)
Counting	20 (16.8%)	10 (13.5%)	17 (18.7%)	3 (10.7%)
Lying still	35 (77.8%)	59 (79.7%)	76 (83.5%)	18 (64.3%)
Reading	13 (28.9%)	31 (41.9%)	35 (38.5%)	9 (32.1%)
Watching TV	16 (35.6%)	23 (31.1%)	30 (33%)	9 (32.1%)
Listening to radio	14 (31.1%)	10 (13.5%)	18 (19.8%)	6 (21.4%)
Using ear plugs	12 (26.7%)	13 (17.6%)	21 (23.1%)	4 (14.3%)
Mobile phones	27 (60.0%)	45 (60.8%)	55 (60.4%)	17 (60.7%)
How often do you wake in the night? (^a^< 4 times a month)	16 (35.6%)	27 (36.5%)	30 (33%)	13 (46.4%)
How long does it usually take to fall asleep again? (^a^> 30 min)	5 (11.1%)	17 (23.0%)	16 (17.6%)	6 (21.4%)
Does a poor night’s sleep (< 6 h) affect you in the following aspects				
Depressed	17 (37.8%)	34 (45.9%)	41 (45.1%)	10 (35.7%)
Anxious	18 (40.0%)	45 (60.8%)	50 (54.9%)	13 (46.4%)
Irritable	27 (60.0%)	49 (66.2%)	58 (63.7%)	18 (64.3%)
Tired	37 (82.2%)	66 (89.2%)	79 (86.8%)	24 (85.7%)
Concentration	33 (73.3%)	59 (79.7%)	72 (79.1%)	20 (71.4%)
Memory	28 (62.2%)	46 (62.2%)	58 (63.7%)	16 (57.1%)
Ability to work	31 (68.9%)	53 (71.6%)	66 (72.5%)	18 (64.3%)
Mean score ± STD	46.7 ± 20.8	49.9 ± 20.1	49.6 ± 20.2	45.4 ± 20.8
*p* value	0.41		0.34	

Data reflect the participants who agreed to the corresponding question (^a^exceptions)

**Table 6:** Comparison of physical activity during the COVID-19 pandemic among males and females in pre-clerkship and clerkship years

Variables	Males *n* (%)	Females *n* (%)	Pre-clerkship *n* (%)	Clerkship *n* (%)
Compared to one year ago, how would you rate your health in general now? (^a^same or worse than one year ago)	32 (71.1%)	55 (74.3%)	70 (76.9%)	17 (60.7%)
The following items are about activities you might do during a typical day				
Vigorous activities, such as running, lifting heavy objects, and participating in strenuous sports	25 (55.6%)	59 (79.7%)	64 (70.3%)	20 (71.4%)
Moderate activities, such as moving a table, pushing a vacuum cleaner, bowling, or playing golf	21 (46.7%)	33 (44.6%)	41 (45.1%)	13 (46.4%)
Lifting or carrying groceries	14 (31.1%)	28 (37.8%)	33 (36.3%)	9 (32.1%)
Climbing several flights of stair	19 (42.2%)	35 (47.3%)	39 (42.9%)	15 (53.6%)
Climbing one flight of stairs	12 (26.7%)	18 (24.3%)	21 (23.1%)	9 (32.1%)
Bending, kneeling, or stooping	16 (35.6%)	17 (23.0%)	24 (26.4%)	9 (32.1%)
Walking more than a mile	19 (42.2%)	32 (43.2%)	42 (46.2%)	9 (32.1%)
Mean score ± STD	43.9 ± 25.9	46.8 ± 21.7	45.9 ± 22.5	45.1 ± 26.2
*p* value	0.51		0.88	

Data reflect the participants who disagreed to the corresponding question (^a^exceptions)

### Scoring Method

The scoring methodology utilized involved assigning a score of 1 for selecting "agree" on the scale and 0 for selecting "disagree". A higher score within the general information category indicated a greater awareness of COVID-19-related content. The scores were categorized into two groups, distinguishing levels of knowledge: those scoring above the mean were classified as having high knowledge, while those scoring below the mean were categorized as having low knowledge. Similarly, an elevated score within the social media category denoted increased use. Conversely, a higher score within the mental and physical health category indicated a negative impact on health. The total scores were calculated for each participant and then divided by the total number of questions within each category. These resulting scores were subsequently multiplied by 100 to derive a percentage score ranging from 0 to 100%. Mean and standard deviation were computed separately based on gender and academic year utilizing these percentage scores.

### Study Population and Sample Size

The study was conducted at Alfaisal University, a private institution located in Riyadh, Kingdom of Saudi Arabia, with an approximate total student population of 1200 at the time of survey distribution. Utilizing a convenience sampling method, we distributed the questionnaire to students via official email and various social media platforms, including WhatsApp and Instagram. Subsequently, we received a total of 146 responses, which represented approximately 12% of the total student population. After excluding participants who self-reported pre-existing psychological conditions prior to the COVID-19 pandemic, 119 responses met the eligibility criteria for inclusion in this study, accounting for approximately 9% of the student population.

### Ethical Considerations

Ethical approval was obtained from the Alfaisal University Institutional Review Board (approval number IRB20091), and the research team followed the ethical guidelines set by the Saudi National Committee of Bioethics (NCBE) and the Research Policies & Procedures of Alfaisal University. Anonymity and confidentiality were ensured by not collecting any identifying data, and the research team only had access to the survey responses.

### Statistical Analysis

The data extracted from the online survey were handled independently, ensuring the exclusion of confidential details. Manual filtering was performed to adhere to the predefined inclusion criteria, and subsequently, the data were analyzed using IBM SPSS Statistics for Windows, Version 28.0 by IBM Corp. Descriptive statistics were employed to present the overall characteristics of the study population, including frequencies and percentages for categorical variables, as well as means and standard deviations for continuous variables. Bivariate analysis was conducted using chi-square test for categorical and binary factors, while independent t-tests were employed for continuous variables, following a normality check using Kurtosis. Pearson correlation was utilized to examine the relationship between independent and dependent variables. Statistical significance was determined using a *p* value of less than 0.05.

## Results

### Demographics

A total of 146 responses were received, and after excluding participants with self-reported pre-existing psychological conditions prior to COVID, we analyzed 119 responses. Out of these 119 participants, 45 were male and 74 were female. In terms of academic years, 91 participants were in years 1–3 (pre-clerkship), while 28 participants were in years 4–5 (clerkship).

### General Information Awareness About COVID-19

Table 1 presents the participants' responses regarding various aspects of COVID-19 knowledge. The results from the statistical analysis did not show any significant relation for the general knowledge about COVID-19 between gender groups (*p* = 0.75) or academic groups (*p* = 0.69).

### Social Media Use Among Alfaisal Students

The overall mean score for social media use among the respondents was 62.8 ± 21.5. Table 2 displays the participants' responses regarding social media use during lockdown. The results indicated that female respondents exhibited slightly higher use with a mean score of 66.8 ± 20.0 compared to males with a mean score of 56.3 ± 22.4 (*p* = 0.01). However, there was no significant difference among the academic years (*p* = 0.51).

### Mental Health Among Alfaisal Students

The overall mean score for mental health among the respondents was 40.7 ± 29.4. Table 3 presents the participants' responses regarding various aspects of mental fitness. The findings revealed that females had a significantly higher mental health score than males, with a mean score of 45.2 ± 29.0, compared to males with a mean score of 33.3 ± 29.1 (*p* = 0.03). However, there was no significant difference among the academic years (*p* = 0.59).

### Physical Health Among Alfaisal Students

Overall physical health was assessed through diet, sleep patterns, and physical activity. The overall mean score for physical health among the respondents was 49.6 ± 14.4. The mean score for diet among the respondents was 97.5 ± 15.7. Table 4 presents the responses related to fast food, soda, and fruit intake. The mean score for sleep patterns among the respondents was 48.7 ± 20.3. Table 5 presents the participants' responses regarding changes in their sleep patterns during lockdown. The mean score for physical activity among the respondents was 45.7 ± 23.3. Table 6 presents the participants' responses regarding changes in their physical activity patterns during lockdown.

No significant differences were found among female and male respondents in terms of physical health, diet, sleep, or physical activity. Similarly, the results for physical health, diet, sleep, and physical activity did not show any significant differences between clerkship and pre-clerkship.

### Correlation Between Social Media Use and Physical/Mental Health

The study demonstrated a notable correlation of 0.30 (*p* < 0.001) between social media use and mental health across all participants, indicating a low positive correlation and suggesting a potential adverse effect on mental health as social media use increased (Table 7). Similarly, a significant correlation of 0.33 (*p* < 0.001) was found between social media use and physical health among all participants, underscoring a potential adverse impact on physical health as social media use increased.

**Table 7:** Correlation coefficients of social media use with mental and physical health by gender and academic year

	Social media and mental health	Social media and physical health
All	0.302 (0.001)	0.332 (< 0.001)
*Gender*		
Males	0.191 (0.21)	0.196 (0.20)
Females	0.321 (0.01)	0.399 (< 0.001)
*Academic year*		
Pre-clerkship	0.263 (0.01)	0.343 (0.001)
Clerkship	0.420 (0.03)	0.292 (0.13)
*COVID awareness*		
Low knowledge^a^	0.292 (0.02)	0.350 (0.01)
High knowledge^a^	0.338 (0.01)	0.328 (0.01)

^a^Low knowledge defined by a score < 78, whereas high knowledge was defined by a score of ≥ 78

Furthermore, when considering the awareness of COVID-19 (Table 7), for students with knowledge scores lower than 78, a low positive correlation of 0.29 (0.02) was observed between social media use and mental health. In contrast, students with knowledge scores equal to or exceeding 78 displayed a slightly higher correlation of 0.34 (0.01), suggesting that individuals with a more comprehensive understanding of COVID-19 had a more negative impact on their mental health as their social media use increased. Regarding the relationship between social media use and physical health, irrespective of awareness levels, a similar correlation was identified (Table 7).

#### Gender-Based Analysis

Further analysis was conducted to assess the association between social media use and physical and mental health for each gender. Among females, a statistically significant correlation of 0.32 (*p* = 0.01) was found, indicating an adverse impact on their mental health with increased social media use. However, no significant correlation was observed among males (*p* = 0.21).

Regarding physical health, females exhibited an even higher correlation of 0.40 (*p* < 0.001), further emphasizing the negative influence of social media on their physical health. Conversely, no significant correlation was found among males.

#### Analysis by Academic Years

The correlation between social media use and physical and mental health was assessed independently for different academic years, as shown in Table 7. Among pre-clerkship students, a significant correlation of 0.26 (*p* = 0.01) was found, indicating a negative effect on their mental health with increased social media use. Similarly, among clerkship students, a significant correlation coefficient of 0.42 (*p* = 0.03) was observed, suggesting a negative impact on their mental health as well.

Regarding physical health, among pre-clerkship students, a significant correlation of 0.34 (*p* = 0.001) was found, highlighting the adverse impact of social media use on their physical well-being. However, among clerkship students, the association was not found to be significant.

## Discussion

Given the limited initial understanding of the COVID-19 pandemic and its consequences, numerous research efforts were initiated to gain deeper insights into the situation. The main objective of this study was to understand the effects of social media on mental and physical health of medical students at Alfaisal University during the pandemic. The participants were analyzed by gender and academic year, aiming to raise awareness and educate the general population on how to avoid or mitigate potential negative impacts caused by social media use during COVID-19.

Our study revealed that males had a higher overall awareness of this data compared to females. However, when examining specific aspects, females demonstrated greater awareness of symptoms (70.95%) and ventilator use (66.2%). Additionally, when comparing academic years, clerkship participants exhibited higher overall awareness, while pre-clerkship students showed greater knowledge of transmission (85.27%) and prevention (83.74%).

Regarding social media use during the COVID-19 lockdown, females reported using it more for purposes, such as feeling preoccupied, escaping from problems, studying, and staying in touch with relatives, compared to males. On the other hand, males showed a higher use of social media for creating their social identity (40%) compared to females. Among academic years, pre-clerkship students exhibited higher use in these aspects than clerkship students. However, clerkship students reported more unsuccessful attempts to curb their social media use (71.4%).

Based on our study's outcomes, compelling evidence emerges concerning the negative impact of social media use on both physical and mental health, particularly among university female students and across various academic years. The findings distinctly reveal a substantial negative influence of social media on the mental well-being of female students compared to their male counterparts. This aligns with previous research indicating a higher prevalence of mental stress among women [9]. Moreover, our study highlighted a significant adverse effect on dietary choices, sleep patterns, and physical activity levels of pre-clerkship students compared to their peers in clerkship. Similar research in India also showed a negative impact on sleep cycles and physical activity among first year MBBS students due to excessive social media use [10].

These results accentuate the pressing need for targeted interventions and educational initiatives to cultivate healthier social media habits and bolster the overall well-being of individuals, particularly during times of crisis. They underscore the critical importance of promoting informed and responsible social media use while actively raising awareness about its potential negative effects on well-being.

According to previous research, it was observed that the COVID-19 pandemic had a significant impact on mental health [16]. Another study found that social media use played a crucial role in the development of panic related to the COVID-19 epidemic, particularly affecting female respondents more than males [11]. Consistent with these findings, our own research demonstrated that social media use had a considerably negative effect on the mental health of female students compared to male students. Our study revealed that a substantial 83.8% of females reported feeling preoccupied with the internet, while 66.2% experienced restlessness, moodiness, depression, or irritability when attempting to reduce or discontinue their internet use. In contrast, 40% of males expressed a diminished interest or pleasure in activities. These outcomes underscore the unique challenges faced by females in managing their mental well-being in relation to their engagement with social media.

Among academic years, the results indicated that the use of social media increased, and there was an adverse impact on the mental health of both pre-clerkship and clerkship years. Also, our findings revealed that 86.8% of students in pre-clerkship years reported staying on their phones for longer than intended, while 57.1% of students in clerkship years had difficulties concentrating on tasks, such as studying.

Existing research has indicated that higher levels of social media use among this student population are associated with lower levels of sports participation, reduced happiness, and increased socioemotional difficulties. Moreover, the use of smartphones or mobile phones at night has been linked to disrupted sleep patterns among adolescents, and frequent social media use at night is associated with perceived insufficient sleep [12]. A previous review study also reported lower levels of physical activity among adolescents during the lockdown, along with a higher proportion of time spent on digital screens [17].

Between physical health and social media use, our study analyzed that there is a correlation of 0.34 (0.001) among pre-clerkship, significantly suggesting that as the use of social media increased, there was harmful impact on their physical health. In terms of eating habits, clerkship students reported excellent habits (100%) compared to pre-clerkship students. Regarding sleep changes, pre-clerkship students experienced a more negative impact, with 72.5% reporting taking more time to fall asleep. When it comes to physical activity, such as climbing stairs and lifting objects, pre-clerkship students indicated a more negative effect than clerkship students, with 76.9% stating that it was worse than a year ago.

When considering gender, females had the association of 0.4 (< 0.001), showing a significant harmful impact on physical health as the use of social media increased. Females reported better eating habits (98.6%) compared to males. However, females also experienced a negative impact on sleep, with 75% reporting taking more time to fall asleep. Moreover, females had a more negative effect on physical activity, with 74.3% stating that it was worse than a year ago, compared to males.

Acknowledging the limitations inherent in our study is crucial. First, the relatively small sample size we worked with may impact the generalizability of our results. Another constraint lies in our study's specific focus on the Alfaisal student cohort, potentially limiting its representation of the broader Saudi population. Future research incorporating a larger and more diverse sample size is imperative to gain a deeper understanding of the true impact of social media.

In conclusion, this study highlights the significance of understanding the effects of social media on the mental and physical health of individuals, particularly during challenging times like the COVID-19 pandemic. By examining the experiences of medical students at Alfaisal University, we have shed light on the potential impacts of social media use and emphasized the importance of raising awareness and providing support to mitigate any negative effect. Our findings contribute to the growing body of knowledge regarding the impacts of social media on mental and physical health, emphasizing the need for informed interventions and strategies to promote a healthy balance in social media use. By addressing these issues, we can strive to foster a more positive and supportive online environment for individuals, both during and beyond times of crisis. Future research should aim to overcome this study’s limitations and include diverse populations to further enrich our understanding of the complex relationship between social media and health outcomes.

## Data Availability

The data of the study, including the code used in the analyses, are available from the corresponding author upon reasonable request.
